# Efficient method for site-directed mutagenesis in large plasmids without subcloning

**DOI:** 10.1371/journal.pone.0177788

**Published:** 2017-06-02

**Authors:** Louay K. Hallak, Kelly Berger, Rita Kaspar, Anna R. Kwilas, Federica Montanaro, Mark E. Peeples

**Affiliations:** 1 Center for Vaccines and Immunity, The Research Institute at Nationwide Children’s Hospital, Columbus, Ohio, United States of America; 2 First Biotech Inc., Athens, Ohio, United States of America; 3 Heidelberg College, Tiffin, Ohio, United States of America; 4 Center for Gene Therapy, The Research Institute at Nationwide Children’s Hospital, Columbus, Ohio, United States of America; 5 Department of Pediatrics, The Ohio State University College of Medicine, Columbus, Ohio, United States of America; 6 Integrated Biomedical Sciences Graduate Program, The Ohio State University College of Medicine, Columbus, Ohio, United States of America; University of Helsinki, FINLAND

## Abstract

Commonly used methods for site-directed DNA mutagenesis require copying the entire target plasmid. These methods allow relatively easy modification of DNA sequences in small plasmids but become less efficient and faithful for large plasmids, necessitating full sequence verification. Introduction of mutations in larger plasmids requires subcloning, a slow and labor-intensive process, especially for multiple mutations. We have developed an efficient DNA mutagenesis technique, UnRestricted Mutagenesis and Cloning (URMAC) that replaces subcloning steps with quick biochemical reactions. URMAC does not suffer from plasmid size constraints and allows simultaneous introduction of multiple mutations. URMAC involves manipulation of only the mutagenesis target site(s), not the entire plasmid being mutagenized, therefore only partial sequence verification is required. Basic URMAC requires two PCR reactions, each followed by a ligation reaction to circularize the product, with an optional third enrichment PCR step followed by a traditional cloning step that requires two restriction sites. Here, we demonstrate URMAC’s speed, accuracy, and efficiency through several examples, creating insertions, deletions or substitutions in plasmids ranging from 2.6 kb to 17 kb without subcloning.

## Introduction

A number of DNA modification techniques involve rapid and efficient site-directed DNA mutagenesis (SDM) developed in the 1990’s, soon after the invention of polymerase chain reaction (PCR) [1]. Most SDM techniques make use of one version or another of inverse PCR mutagenesis. Inverse PCR was developed by Hemsley *et al*. [2] and later improved by the use of a proof-reading DNA polymerase, such as Vent [3], and an enzymatic step to remove background template [4]. Another fast mutagenesis method developed by Papworth *et al*. [5] uses a primer extension design to copy the plasmid, generating staggered nicks that are repaired by bacteria after transformation. These and other SDM methods have been commercialized in DNA mutagenesis kits such as ExCite and QuikChange (Stratagene, La Jolla, CA), and Phusion Site-Directed Mutagenesis (New England BioLabs, Ipswich, MA). Because these techniques rely on copying the entire DNA plasmid with primers containing the desired mutation, they generally work best for plasmids under 3.1 kb in size [6]. This approach has inherent limitations including the difficulty of copying large plasmids, an increased chance of encountering too-high or too-low GC contents that slow or stop PCR reactions, and the introduction of unwanted mutations in the plasmid due to polymerase errors. Introducing a mutation in plasmids larger than 8 kb usually requires subcloning a section of the plasmid containing the site of mutagenesis into smaller cloning vectors to make the SDM possible. Subcloning is an inherently slow process involving restriction enzyme digestion, ligation, transformation, colony formation and selection, DNA isolation, sequence verification, and excision of the mutated DNA sequence from the subclone and its insertion into the original plasmid. All but the final insertion step are avoided in URMAC.

URMAC employs a minimalistic approach in which PCR reactions are performed on the smallest possible portion of a large plasmid that contains the mutagenic target site flanked by unique restriction sites. This approach significantly improves the rate of PCR success and the quality of the product. In this study, we provide several examples of the applicability of URMAC for deletion, insertion, or substitution of DNA sequences in plasmids ranging in size from 2.6 kb to 17 kb.

## Results

### General description of the URMAC method

**Fig 1:**
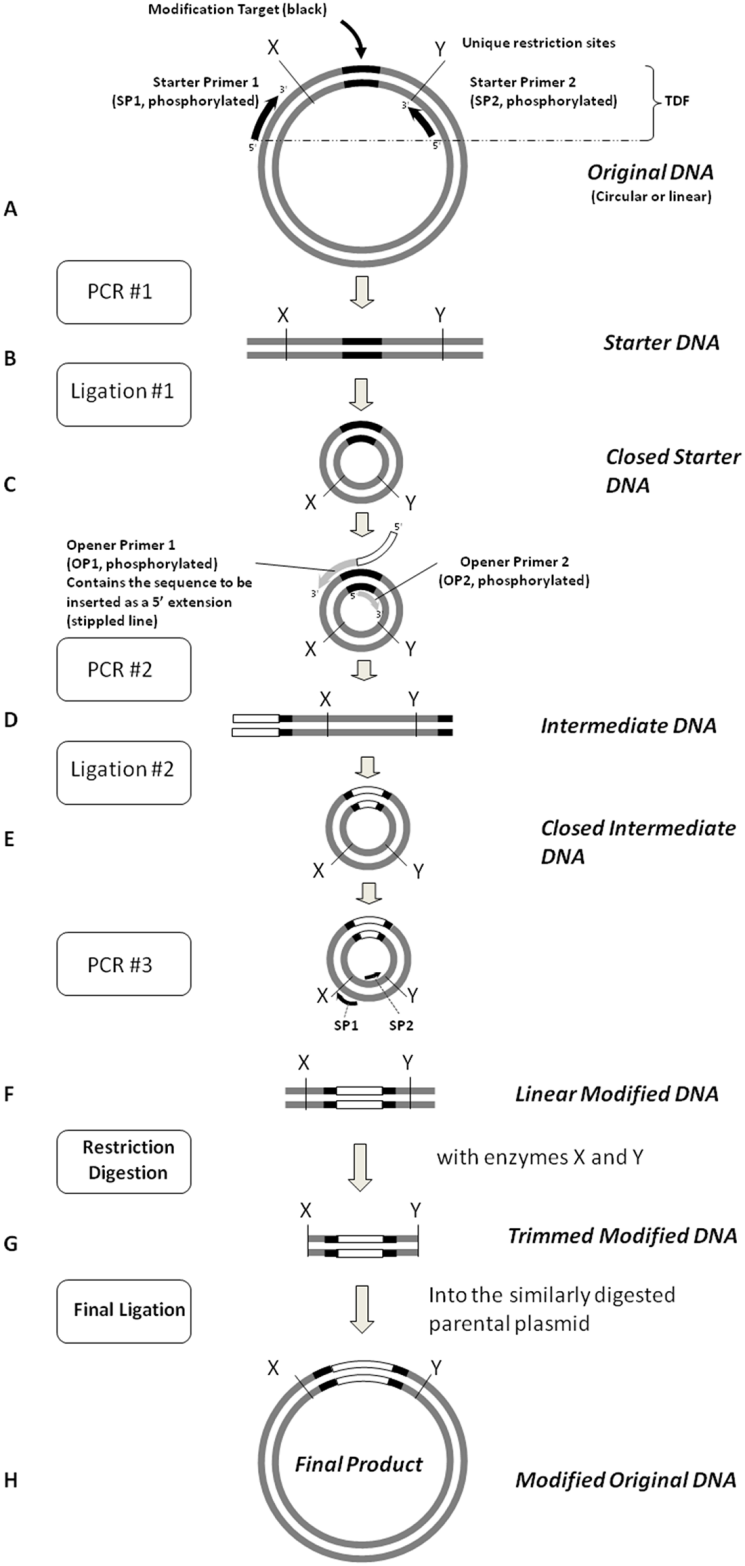
The URMAC method. This illustration depicts a hypothetical insertion within a *Modification Target* (black lines) in the *Original DNA* plasmid. PCR #1 generates the *Starter DNA* copy of the *Modification Target* including the flanking unique restriction sites, X and Y. It is produced by a thermostable DNA polymerase using the *Starter Primers*, SP1 and SP2 (black arrows). The *Starter DNA* is circularized with T4 DNA ligase to generate the *Closed Starter DNA*. *The Closed Circular DNA* serves as the template for PCR #2, directed by the *Opener Primers*, OP1 and OP2, to produce the mutated *Intermediate DNA*. In this illustration, OP1 has incorporated an insertion mutation by having the sequence of interest (depicted as an open box) attached to its 5′ terminus. The *Intermediate DNA* is circularized with T4 DNA ligase. The SP1 and SP2 primers are used in the enrichment PCR step to amplify the *Linear Modified DNA*. *The Linear Modified DNA*, and the original plasmid are digested with the restriction enzymes that cleave at the unique restriction sites, X and Y, and the appropriate fragments are ligated to produce the *Modified Original DNA*.

URMAC relies on the simple ability of DNA ligation to turn a linear PCR product generated from a plasmid template into a circular DNA that can be opened at a second site by amplification with primers containing the desired mutation(s), circularized by ligation again, and amplified with the original primers to reproduce the original DNA containing the desired mutation(s). The same reaction steps can be used to insert, delete, or substitute any number of DNA nucleotides. Two sets of primers are used in URMAC, the *Starter Primers* and the *Opener (Mutagenic) Primers*. These primers are 5′ phosphorylated so that they can participate in the subsequent ligation step. The *Starter Primers* are first used to amplify the Modification Target sequence and again for the final enrichment PCR step. The *Opener Primers* are used to introduce the mutation of interest. The steps involved in URMAC are illustrated in Fig 1 and described below.

#### PCR #1

The *Modification Target*, a small portion of the plasmid containing the sequence to be modified, is selected for amplification by PCR. The positions of the *Starter Primers* (SP1 and SP2) are chosen such that the amplified *Starter DNA* includes the closest, but at least 150 bp apart, unique restriction sites (X and Y) in the plasmid flanking the site to be modified. These restriction sites will be used in the final step for insertion of the *Modified DNA* into the parental plasmid.

#### Ligation #1

The *Starter DNA* is circularized in a self-ligation reaction to generate the *Closed Starter DNA*.

#### PCR #2

The *Closed Starter DNA* is opened at the site of the desired mutation by inverse PCR using the *Opener (Mutagenic) Primers* (OP1 and OP2) facing opposite directions from the opening site to generate the linear *Intermediate DNA* containing the mutation(s) at its termini. Sequences can be: deleted at the site of opening by moving the primers apart; inserted by adding nucleotides to the 5′ terminus of one or both of the primers; or mutated by changing one or more nucleotides in one or both primers. Any combination of deletion, insertion or substitution can be designed into the *Opener Primers*.

#### Ligation #2

The *Intermediate DNA* is circularized by self-ligation to generate the *Closed Intermediate DNA*. The desired mutation is now in place and the fragment can be excised and ligated into the original plasmid.

#### Optional enrichment PCR Step

The *Closed Intermediate DNA* can be amplified by the same pair of *Starter Primers* that were used to amplify the *Starter DNA* in the first step (SP1 and SP2) to generate the *Linear Modified DNA*. This PCR step increases the number of DNA molecules with desired modifications for ligation into the original plasmid.

#### Restriction digestion and insertion

Both the *Linear Modified DNA* and the original plasmid are digested with the unique restriction enzymes identified in the first step (X and Y in Fig 1), and purified. The final DNA with the modifications sequence is ligated into the parental plasmid to produce the final product containing the desired mutation(s). By the end of the final PCR enrichment step, the amount of the Original DNA carryover from the first PCR reaction step is insignificant due dilution factors. Optionally, the addition of 1unit *DpnI* restriction enzyme to the first ligation step completely removes any potential carryover of the full Original DNA plasmid during the URMAC procedures. The success rate of obtaining the correct clones after the final cloning step is normally over 95%.

### Validation of the method: Introducing insertion, deletion and substitution mutations

**Fig 2:**
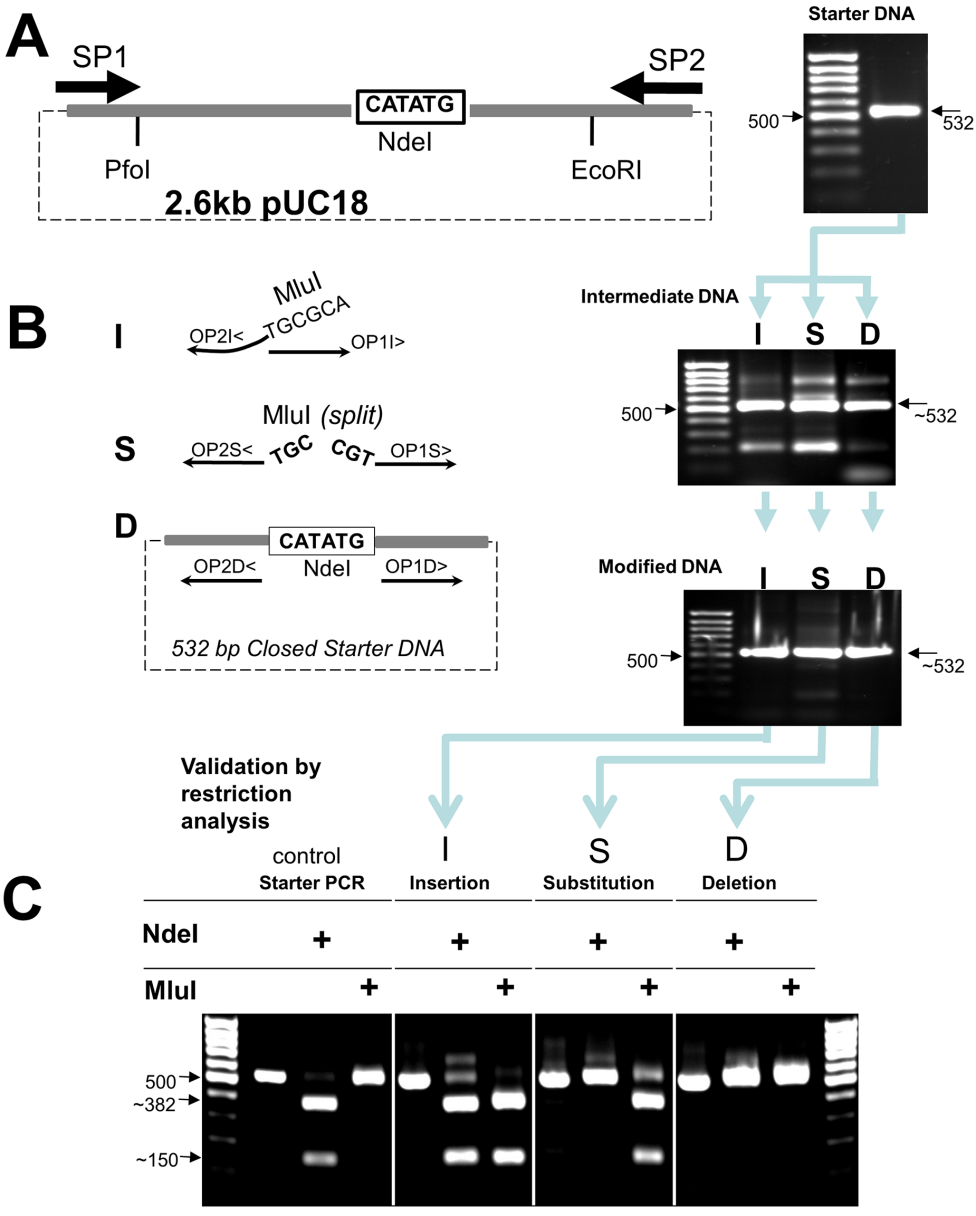
Validation of the URMAC method by insertion (I), substitution (S), or deletion (D) of some restriction sites in pUC18 plasmid. (A) Illustration of the *Modification Target* (NdeI restriction site) relative to the flanking restriction sites and location of the *Starter Primers* SP1 and SP2. After the first PCR, the *Starter DNA* migrated as expected, 532 bp on a 1% agarose gel (photo, arrow at right). A 100 bp DNA size ladder is shown in left lane for comparison. (B) Diagram of the strategy for I, S, or D using the *Closed Starter DNA* circularized from the PCR product in (A) as template and the *Opener/Mutagenic Primers*. The top photo shows the PCR product, *Intermediate DNA*, which contained the mutations. The bottom photo shows the *Modified DNA* after enrichment PCR step using SP1 and SP2. (C) Validation of URMAC mutagenesis for the three different types of mutations by restriction analysis. Fig 2C shows bands of expected DNA fragment size after digestion with respective restriction enzymes. In the control Starter PCR lane, only DNA treated with NdeI enzyme, cut the DNA into two fragments of 382 & 150 bp. Untreated DNA or DNA treated with MluI remained at the full size of 532 bp. In the Insertion lane, both NdeI and MluI cut the DNA at the expected sizes of 382 & 150 for NdeI and 383 & 149 for MluI. In the Substitution lane, only MluI cut the DNA producing the expected 383 & 149 bp bands. In the Deletion lane, none of the enzymes cut the DNA, leaving the bands at their original Modified DNA size.

For validation of the URMAC method, we used pUC18, a widely available plasmid, as a target to test URMAC by either removing or adding a restriction site. We performed all three different types of DNA mutagenesis using the same *Closed Starter DNA* from the first PCR and ligation reactions as a template (Fig 2A).

We inserted the recognition sequence of a MluI restriction enzyme next to that of the native NdeI, substituted the NdeI recognition sequence with that of the MluI (that is simultaneous deletion of NdeI and insertion of MluI recognition sequences), or deleted the NdeI recognition sequence, all by altering the 5′ ends of the OP1 and OP2 primers or positioning them appropriately (Fig 2b, and Table 1).

**Table 1:** List of primers.

Primer Name	Sequence
Primer sets for pUC18 deletion, insertion, and substitution	
*SP1*	ACACATGCAGCTCCCGGAGA
*SP2*	CTCACTCATTAGGCACCCCAGG
*OP1D>*	CGGTGTGAAATACCGCACAGAT
*OP2D<*	GTGCACTCTCAGTACAATCTGC
OP1S>	***CGT***CGGTGTGAAATACCGCACA
OP2S<	***CGT***GTGCACTCTCAGTACAATC
OP1I>	CATATGCGGTGTGAAATACCGCAC
OP2I<	***ACGCGT***GTGCACTCTCAGTACAAT
H glycoprotein mutagenic primers for the LDV motif	
SP1-H>	TTTGTCATGTTTCTGAGCTTG
SP2-H<	CAAGTGAGATCTCTGAAGTCG
Common Reverse primer OP2-H<	ATTGGTGCTGAGGCTTTTATG
Leu to Ala H-OP1-LΔA>	*GC*AGATGTAACTAACTCAATC
Asp to Ala H-OP1-DΔA>	CTAG*C*TGTAACTAACTCAATCGA
Asp to Glu H-OP1-DΔE>	CTAGAAGTAACTAACTCAATCGAG
Val to Ala H-OP1-VΔA>	CTAGATG*C*AACTAACTCAATCGAGCA
F Glycoprotein mutagenic primers for the LDV motif	
SP1-F>	CCAAGTATGTCGCAACCCAAG
SP2-F<	ACTCCTCAATATCTGGTCCGA
Reverse Primer OP2-F<	CCTCTCCAATGATATGGGAGG
Leu to Ala F-OP1-LΔA>	*GC*GGACGTAGGGACAAATCT
Asp to Ala F-OP1-DΔA>	TTGG*C*CGTAGGGACAAATCTGGG
Asp to Glu F-OP1-DΔE>	TTGGA*G*GTAGGGACAAATCTGGGG
Val to Ala F-OP1-VΔA>	TTGGACG*C*AGGGACAAATCTGGGGAA
Primer set for substitution mutation A1043G in the MD cDNA	
SP1-MD>	ATAGTCATAGGCCAGACC
SP2-MD<	CCACAGTAATCTGCCTCTTC
OP1-MD>	CTATTCTCAACAGATC**G**CGGT
OP2-MD<	CAGTCTAGCACAGGGATATGA
Primer set for deletion of the M2-1 or M2-2 ORF from the RSV replicon cDNA	
SP1-RSV>	ACTTGTATCGTCGCCATCGG
SP2-RSV<	AGAAACGTAGTCCTGATAAC
OP1-RSV<	ATTTGCCCCAGTTTTCATTTTTAC
OP2-RSV>	CAAATGACCATGCCAAAAATAATGATAC
OP3-RSV<	GTCAGGTAGTATCATTATTTTTG
OP4-RSV>	CACCACATCGTTACATTATTAATTC

* Primers in this table are modified by the addition of a phosphate group at their 5′ ends. Abbreviations: SP, Starter Primer; OP, Opener Primer; I, insertion; D, deletion; S, substitution; MD, muscular dystrophy; and RSV, respiratory syncytial virus. The capital H in primer names indicates the primer was designed for mutagenesis in the measles H glycoprotein DNA sequence in pCG-H plasmid and the F in the fusion glycoprotein DNA sequence in the pCG-F plasmid. The “>” sign = forward and the “<” sign = reverse primer orientation. Underline: native NdeI recognition sequence. Bold, italics: inserted MluI recognition sequence. Bold and underlined G in OP1-MD reflects a substitution of A to G.

Since the *Starter DNA* was only 532 bp long (Fig 2A), roughly 20% of the full plasmid size, it was amplified in 60 minutes, more quickly than the whole plasmid would have been. The mutagenesis steps followed the basic URMAC steps illustrated in Fig 1.

Successful mutagenesis was verified by restriction analysis (Fig 2c). The PCR-enriched *Linear Modified DNA* from each mutagenic reaction was digested with NdeI, which cuts once, except when the NdeI site was replaced by substitution (S) or removed by deletion (D). MluI cuts only the inserted (I) or substituted (S) MluI *Linear Modified DNA*.

### URMAC mutagenesis in plasmids with complex GC regions

PCR reactions on GC-rich templates are problematic. Additives such as DMSO, glycerol, formamide, PEG and other organic compounds [7–10] can help to overcome some of the problems associated with PCR on GC-rich templates, However GC, complexities remains a problem that can be avoided by limiting the mutagenesis to smaller regions of the target DNA rather amplifying the full plasmids. In this experiment, we aimed to introduce substitution mutations in two expression plasmids, pCG-H (6,669 bp) and pCG-F (6,411 bp), having GC-rich regions that had failed in previous inverse PCR mutagenesis attempts in our laboratory. At 40-nucleotide resolution scanning (see methods), the plasmids contained a 79.5% GC region in pCG-H and 85.4% in pCG-F. We avoided the need to amplify these GC regions which would have been necessary if we had used inverse PCR mutagenesis, by amplifying and manipulating only the region of interest by URMAC.

The pCG-H and pCG-F plasmids contain the attachment (H) and fusion (F) glycoproteins of Edmonston B strain of measles virus (MV) [11]. Together, these two viral glycoproteins enable the MV virion envelope to attach to and fuse with the target cell membrane to initiate infection. When the H and F proteins are co-expressed on the cell surface during infection or following transfection, they interact with cellular receptors on neighboring cells causing cell-cell fusion resulting in the formation of syncytia. Based on this fusion phenomenon, we tested the functional ability of mutant H and/or F with a modified integrin-binding Leu-Asp-Val (LDV) motif to induce syncytia in two cell lines, Vero and BKH-21. In Vero cells, our positive control, the MV glycoproteins can use the cell surface CD46 molecule as a receptor to initiate fusion independent of interaction with the LDV motif. However, BHK-21 hamster cells do not express CD46, but they express the integrins that interact with the LDV motifs on viral H and F proteins. To determine whether or not the LDV motif in either the H or F glycoprotein is critical for fusion, we used URMAC to replace the central Asp (D) with the similar amino acid, Glu (E), to create an H protein variant, D_79_E, and an F protein variant, D_461_E.

To perform this mutagenesis, we designed a pair of *Starter Primers* (Table 1) to amplify the region encoding the LDV motif in the F gene in pCG-F, and another pair for the H gene in pCG-H, including appropriate native restriction sites in the amplicons. The substitution reaction steps were performed as described above for pUC18. After generating the final plasmids that carry the desired modifications, the DNA of 5 clones for both mutants was sequenced at the mutation sites. All 10 sequenced clones contained the correct mutations. The biological significance of the mutations was investigated by performing a fusion assay(data not shown).

**Fig 3:**
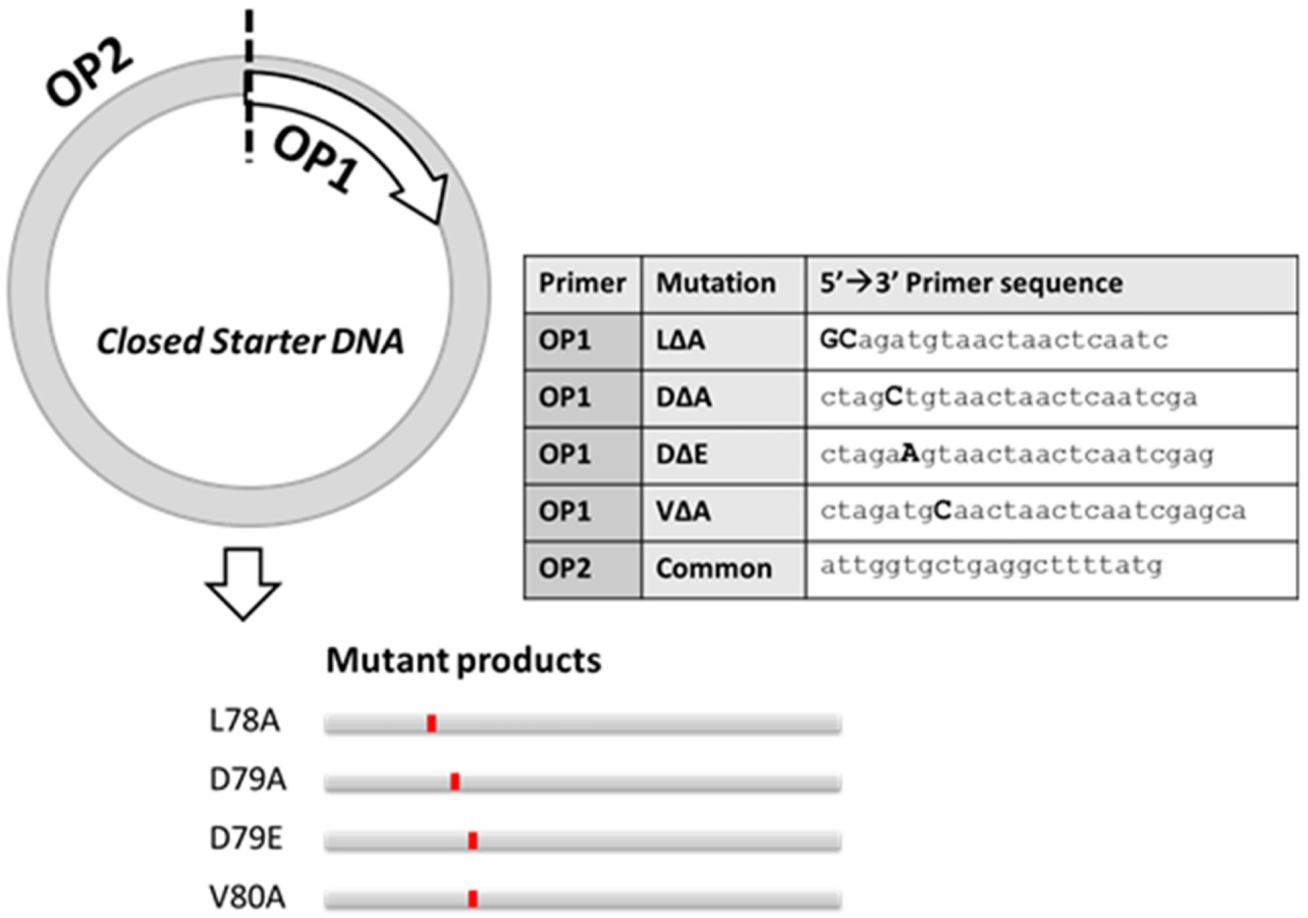
Pairing a variety of mutagenic Opener Primers (OP1 series) with a single common primer (OP2) was used to generate four different variants of the MV H glycoprotein. The OP1 series (Table 1) were used in separate PCR reactions with the same OP2 primer on the same Closed Starter DNA to simultaneously generate four different mutant Linear Intermediate DNA PCR products containing indicated mutations (lower panel). These mutant PCR products were circularized, amplified with the /Starter Primers/ and cloned into the Original DNA plasmid generating four different pCG-H plasmids, each expresses a different MV H glycoprotein variant.

In addition to the D-to-E mutations in pCG-H and pCG-F, we generated several other mutations in the LDV motif of both the F and H proteins using a single *Opener Primer* in conjunction with a series of mutagenic *Opener Primers* on the same *Closed Starter DNA* template. Fig 3B illustrates this approach that generated four different mutations, L78A, D79A, D79E, and V80A, using a single common Opener Primer (OP2) paired with a series of mutagenic Opener Primers (OP1). This approach easily enables any number of mutations to be built into a motif or region by changing the sequence of only one of the primers used in this step. Introducing mutations in the pCG-H and pCG-F took one day for the URMAC biochemical reactions and an additional 3 days to clone the *Linear Modified DNA* into the original plasmids. URMAC did not require extensive optimization since the PCR was used to copy only 10% of the plasmid in this case avoiding regions of high GC content. We conclude from this experiment that URMAC can be easily used to introduce mutations in 6–7 kb plasmids containing regions of widely disparate GC content without resorting to conventional subcloning.

### Introduction of a point mutation into the 11 kb dystrophin cDNA by URMAC

Large plasmids present a challenge for PCR-based mutagenesis methods that require amplifying the full sequence while preventing the introduction of unwanted mutations caused by PCR infidelity. A fragment of the cDNA can be subcloned into a smaller plasmid, mutagenized and the fragment returned to the original plasmid, but this process is time consuming.

As an example, the cDNA for muscle dystrophin open reading frame is approximately 11 kb [12]. Mutations in dystrophin are associated with a spectrum of clinical phenotypes, grouped as “dystrophinopathies” including Duchenne muscular dystrophy (DMD), Becker muscular dystrophy (BMD), and X-linked dilated cardiomyopathy (XLDCM) [13, 14]. DMD and BMD are characterized by progressive skeletal muscle degeneration and development of cardiac disease leading to premature death, with DMD having an earlier childhood onset and a more severe disease progression than BMD. By contrast, XLDCM patients typically only develop severe cardiac disease and as a result, are treated with a cardiac transplant. To study the differential effects of mutations on the function of dystrophin in striated muscles, it is critical to ensure that only the desired mutation is introduced during the mutagenesis process. Given the 11kb size of the cDNA alone, introduction of mutations in dystrophin would require subcloning.

To determine whether URMAC could be a viable and fast substitute for subcloning in such a large plasmid, we sought to introduce a point mutation in the full length dystrophin cDNA. We chose the A1043G missense mutation in exon 9 that results in a Threonine to Alanine substitution at amino acid 279. This substitution is found in one family with very early onset and severe XLDCM [15, 16].

**Fig 4:**
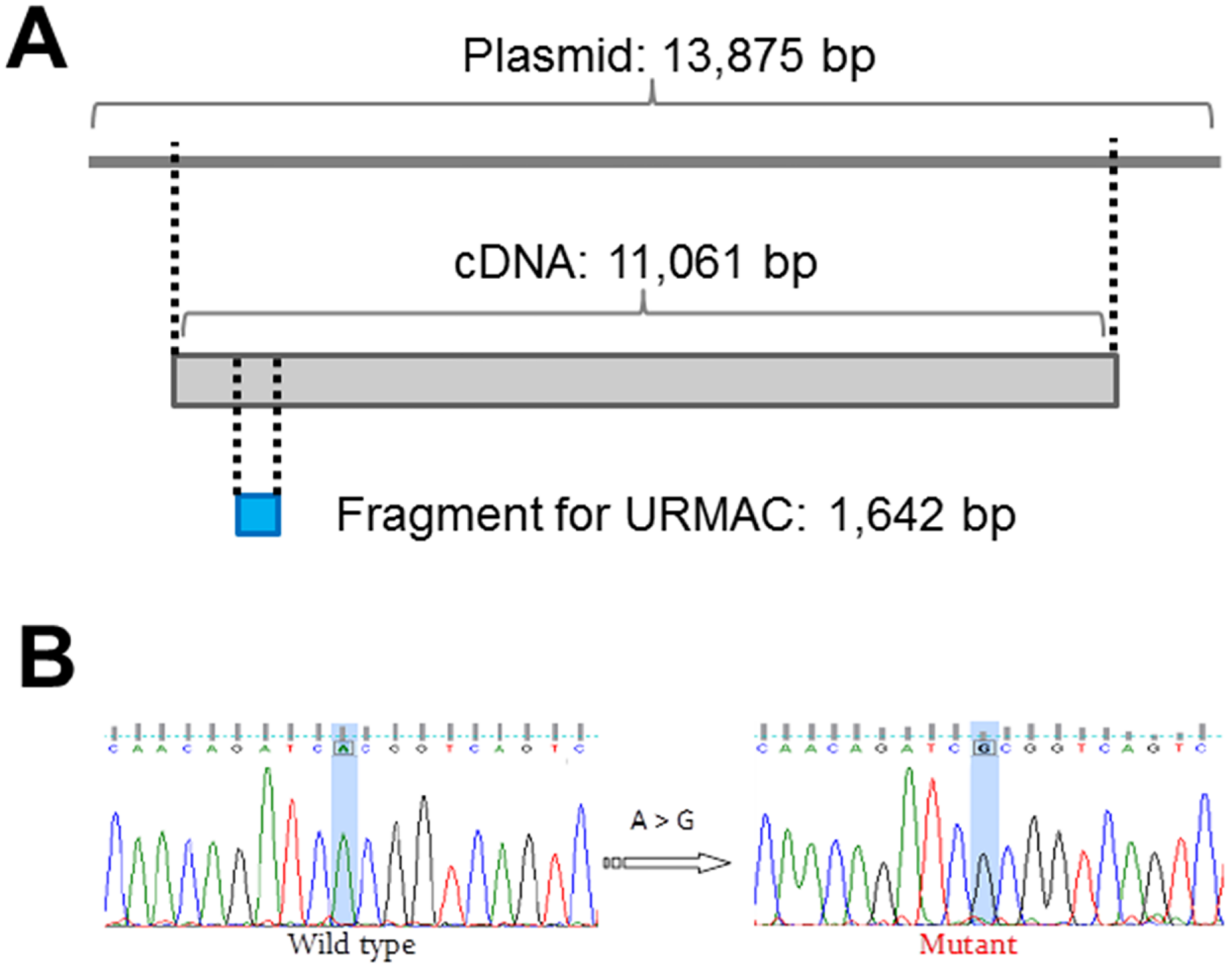
Single base pair substitution in dystrophin using URMAC. (A) Schematic representation of the relative sizes of the original plasmid, the full length human dystrophin cDNA within the plasmid and the final fragment that was isolated for point mutagenesis using the URMAC method. The rest of the dystrophin cDNA sequence was left intact. (B) Sequencing of the mutagenized region after re-ligation into the dystrophin cDNA at the correct position. The desired single A to G substitution was the only mutation found by sequencing the entire dystrophin cDNA.

We started with a commercially available 13.8 kb Gateway entry vector plasmid containing the full length human muscle dystrophin cDNA (11,061 bp). As shown in Fig 4a, the URMAC approach was used on an isolated 1,629 bp region containing exon 9 and two flanking unique restriction enzyme sites (*NsiI* and *SphI*).

This allowed us to modify this short fragment without copying the rest of the cDNA or plasmid sequences. The region to be altered was amplified using the *Starter Primers* (Table 1) to produce the *Starter DNA*. The *Starter DNA* was ligated and amplified with the mutagenic *Opener Primers* (Table 1) containing the A to G mutant sequence to generate the *Intermediate DNA*. Following circularization, the *Starter Primer* pair was used to generate the *Linear Modified DNA*. This entire procedure was completed in 6 hours. The *Linear Modified DNA* was digested with *NsiI* and *SphI* and inserted into the original plasmid. Sequencing of the cDNA region that was isolated for URMAC confirmed the A1043G point mutation (Fig 4B), and sequencing of the entire cDNA, confirmed that this was the only mutation.

Despite the size of the human dystrophin cDNA, introducing a point mutation by URMAC was simple and fast, requiring minimal effort and materials. The entire process from beginning to full sequencing of the final mutagenized plasmid was completed in less than one week.

### Deletion of open reading frames from a large plasmid containing multiple genes

In this example, we used URMAC to delete two open reading frames (ORF) from a 17 kb plasmid containing the human respiratory syncytial virus (RSV) genome from which its three glycoprotein genes had already been deleted and two foreign marker genes inserted by conventional subcloning methods. Transcription of this plasmid in mammalian cells, along with the expression of the 4 viral proteins involved in genome replication and mRNA transcription, initiates continuous intracellular replication of this RNA virus replicon [17, 18]. The aim was to determine whether two of the RSV genes, M2-1 and M2-2, are required for RSV replication and survival.

**Fig 5:**
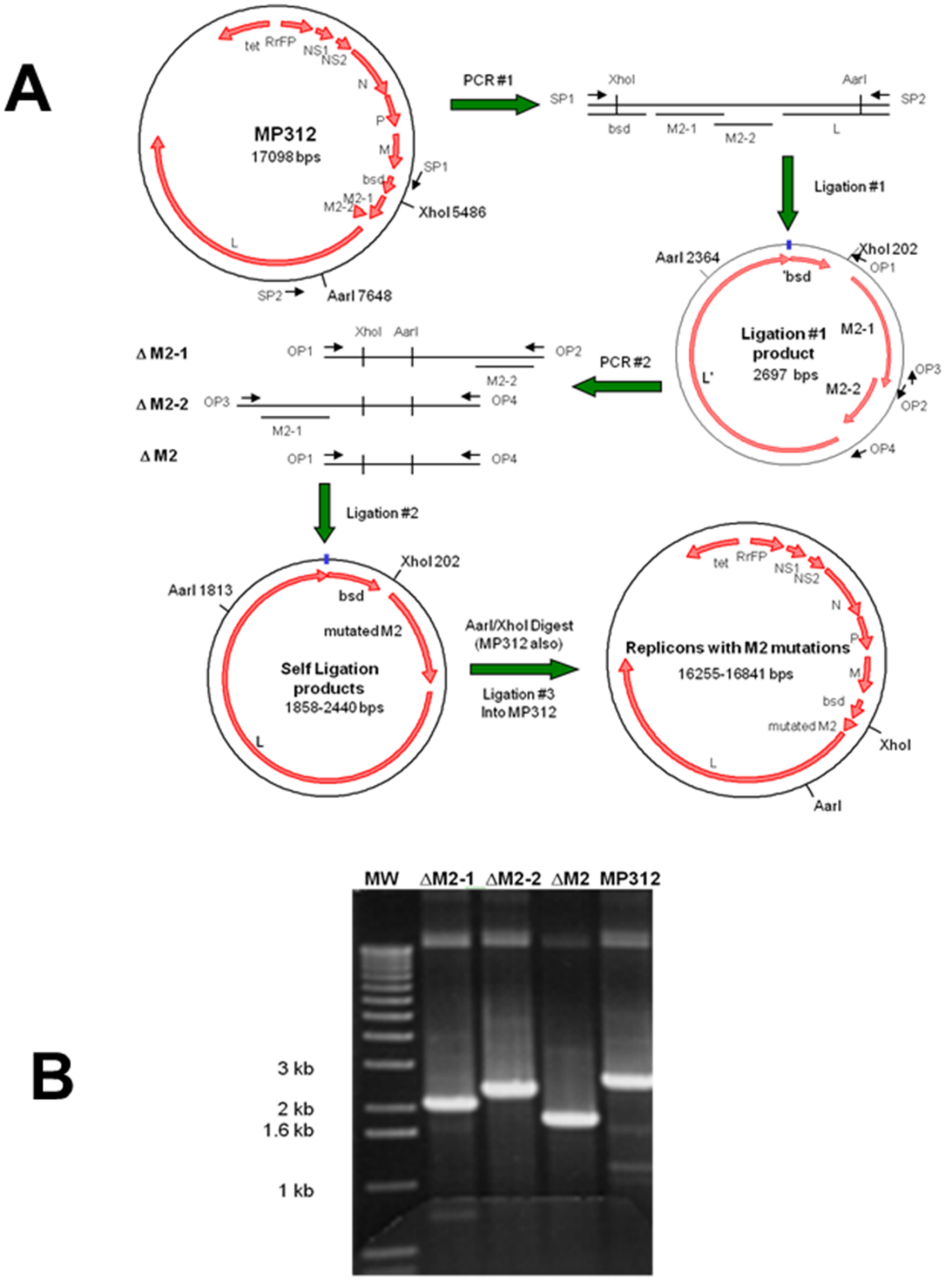
Deletion of the M2-1 or M2-2 ORF, or both from the RSV replicon plasmid. (A) The URMAC scheme used to generate the three M2 deletion replicon constructs. (B) PCR confirmation of the final replicon constructs. Primers SP1 and SP2 were used to amplify the region encompassing all of the desired mutations, by PCR. The targeted deletion region of each replicon plasmid is listed above each lane (ΔM2-1, ΔM2-2, ΔM2). Predicted PCR product sizes for the deletions were 2.1 kb, 2.4 kb, and 1.8 kb, respectively. The comparable product from the parental replicon plasmid, MP312, containing the entire M2 gene was 2.6 kb. MW indicates molecular weight markers.

At 17 kb, this plasmid was too large to be modified by current methods without subcloning. We used the URMAC method to individually delete the M2-1, M2-2 or both ORFs from the replicon cDNA by the scheme shown in Fig 5a.

The *Starter Primers* were designed to flank the M2 gene and the unique *XhoI* and *AarI* sites, and used to produce a 2.7 kb *Starter DNA*. Following ligation, the *Closed Starter DNA* was subjected to the second round of amplification using various pairings of the 4 *Opener Primers* to generate *Linear Intermediate DNAs* with the deletions.

Following ligation, each of the three *Closed Intermediate DNAs* were enriched by a third PCR using the *Starter Primers*. The three resulting *Linear Modified DNAs* were digested with *XhoI* and *AarI* and inserted into the parental plasmid to generate three RSV replicon plasmids with: M2-1 ORF deleted (ΔM2-1), M2-2 ORF deleted (ΔM2-2), and both ORFs deleted (ΔM2). Deletions were confirmed by the size of the PCR products amplified by the *Starter Primers* (Fig 5b) and by sequencing the DNA region between the *XhoI* and *AarI* restriction sites. URMAC enabled the rapid deletion M2-1 and M2-2 from this large plasmid, allowing a quick test of the importance of the M2 genes for RSV genome replication and survival.

## Discussion

The URMAC technique provides several advantages over other existing technologies for DNA mutagenesis. In URMAC, mutagenesis is minimalistic: only the smallest region targeted for mutagenesis in a given DNA sequence/plasmid is subjected to molecular manipulation. For example, in our MV H and F gene mutagenesis experiments, URMAC was applied to only 287 bp and 512 bp of the pCG-H and pCG-F plasmids instead of the entire 6,669 bp and 6,411 bp, leaving more than 95% of the plasmids untouched by the DNA polymerase. This lends several benefits to the URMAC technology: 1) PCR reactions have a higher success rate with small fragments rather than with full-length plasmids; 2) URMAC has a lower chance of introducing polymerase errors than the primer extension SDM method (QuikChange) and inverse PCR because the fragment being amplified is much smaller; 3) regions of any plasmid that are not a direct target for mutagenesis remain untouched by DNA polymerase and therefore do not require sequence verification, a critical consideration when dealing with plasmids containing a very large gene such as the dystrophin gene, or multiple genes such as the RSV replicon; 4) URMAC is very fast compared to conventional SDM requiring subcloning, with an average of a single day to complete URMAC and an additional 3 days to clone the final product into the original plasmid, compared to at least 3–4 weeks required for subcloning; 5) URMAC can dramatically reduce the challenge of high GC-containing plasmids by avoiding PCR amplification of those parts of a plasmid; and 6) URMAC requires less labor and materials and therefore costs less than subcloning.

Furthermore, URMAC is versatile for handling any combination of insertions, deletions or substitutions. While QuikChange is fast in creating a single or double nucleotide mutation in a small plasmid, larger or multiple changes are more difficult. URMAC does not suffer from this limitation since the mutation is inserted at the 5′ end of a short primer rather than in the middle of longer mutagenic primers. In this way, URMAC is similar to the inverse PCR techniques, yet it does not suffer from the size limitation of inverse PCR. Like URMAC, another method, called splice overlap mutagenesis (SOM) [19], requires the availability of restriction sites, but SOM primers must be complementary to the target DNA in both orientations at the joining ends of the two PCR products, limiting control over the primer design and their effectiveness. Since URMAC does not require the joining of PCR products, URMAC does not suffer from this limitation. When individual mutations are required in adjacent regions of the plasmid to generate multiple separate mutants, the *Closed Starter DNA* can serve as a template for all of the mutations by designing different *Opener Primers*. The *Closed Intermediate DNA* can be recycled as *Closed Starter DNA*, once for each mutation.

Although URMAC mutagenesis is not affected by the size of the original plasmid because the actual mutagenesis is performed on only a small region, inserting the final PCR product into the original plasmid does depend on the availability of unique restriction sites. In plasmids of 30 kb or larger, the availability of such restriction sites becomes limited. In this case, an alternative recombineering strategy [20] could be incorporated into the URMAC technique to facilitate the insertion of the mutated sequences back into very large plasmids irrespective of the availability of restriction sites.

In summary, compared to conventional site-directed mutagenesis methods, including those that require subcloning, the URMAC technology is versatile, simple, efficient and cost-effective. It is particularly useful for large plasmids but also works well for small plasmids.

## Methods

### Mutations in pUC18 accession number L09136

All PCRs were carried out using 100 pg pUC18 as a template and 75 pmol of *Starter Primers* 1 and 2 (Table 1) in a total volume of 25 μl containing 1 unit *Pfu* DNA polymerase (Agilent Technologies, Santa Clara, CA) and the appropriate amplification buffer unless otherwise stated. PCRs were performed using the following thermocycling conditions: denaturation at 94°C for 2 min followed by 25 cycles of 94°C, 20 sec; 60°C, 30 sec; 68°C, 1 min, and a final extension at 68°C for 5 min. DNA electrophoresis on 1% agarose gel confirmed that the *Starter DNA* had been produced (Fig 2A). 2.5 μl of the *Starter DNA*, estimated at 50–100 ng, was circularized by self-ligation using 1 unit of T4 DNA ligase in a total reaction mix of 20 μl for 10 min at 20°C. 1 μl of a 1:200 dilution of the *Closed Starter DNA* product in deionized H_2_O was used as a template for the second round of PCR, but this time various pairs of *Opener Primers* (Table 1) were used. 2.5 μl of these products were self-ligated with T4 DNA ligase as above and 1 μl of a 1:200 dilution of the *Closed Intermediate DNA* product was used as a template for the final PCR reaction to create the *Linear Modified DNA*. Successful mutagenesis was confirmed by restriction analysis using NdeI and MluI restriction enzymes (New England Biolabs, Ipswich, MA): 2 μl of *Linear Modified DNA* for each mutation type was incubated with or without 5 units of restriction enzymes for 30 min at 37°C. 5 μl of each reaction was resolved by electrophoresis on a 1% agarose gel. For cloning the modified DNA into the parental DNA, a standard protocol was used, briefly, both the fragment and the plasmid were digested with 5 units of *PfoI* and *EcoRI* separately. The appropriate fragments were gel-purified using QIAquick Gel Extraction kit (Qiagen MiniPrep Kit (Qiagen, Hilden, Germany) and eluted with 30 μl TE buffer. The DNA was measured by Nanodrop^™^ 2000C instrument (Thermofisher, Wilmington, DE). 15 fmol of plasmid’s backbone was mixed with 45 fmol insert and incubated with T4 DNA ligase and ligation buffer for a total of 20 μl and the reaction was incubated for 1 hr at 16°C. The reaction was terminated by incubation at 65°C for 10 minutes. The reaction was then diluted 5-fold in pure H2O. 5 μl of diluted reaction was used to transform 50 μl DB3.1 *E*. *coli* chemically competent cells (Invitrogen). For sequence screening of final clones, the same Starter Primers were used for sequencing the full 500 nucleotide span of the modification target.

### Introducing substitution mutations into the H and F glycoproteins of MV

*Starter Primers* (Table 1) were used to amplify the DNA sequences that include the LVD motifs (5′-CTA GAT GTA-3′ and 5′-TTG GAC GTA-3′) in the pCG-H and pCG-F [11] plasmids containing the H and F glycoprotein genes, respectively, and the flanking, unique restriction sites (*NheI* and *BspEI* for the H gene and *KpnI* and *XcmI* for the F gene). The *Starter DNA* for H was 287 bp and for F was 512 bp. The pCG-H and pCG-F plasmid sizes were 6,669 bp and 6,411 bp, respectively. PCR amplifications and ligations were performed as described for the pUC18 mutagenesis above.

GC region scanning was performed using BioAnnotator module of Vector NTI version 11 (Invitrogen, Carlsbad, CA). Briefly, full DNA sequences of pCG-H or pCG-F plasmids were loaded into the BioAnnotator program, then subjected to GC% analysis under the Analyze Selected Molecule tab. The GC% scanning window was left at the default of 40 nucleotide under the Select Window tab. Percent of GC% contents of each plasmid were noted at the highest peaks of the histogram.

### Mutagenesis of muscular dystrophy gene

A modified Gateway entry vector (pENTR223.1, Clone ID: 40080544) containing the full-length human muscle dystrophin cDNA (11.061 kb) was obtained from the ORFeome Collaboration-OCAB (http://www.orfeomecollaboration.org/html/index.shtml). The sequence is deposited in NCBI under accession number BC111587. The Pfu DNA Turbo polymerase (Stratagene) was used for all PCRs. Thermocycling conditions for the *Starter Primers* (Table 1) for the first and third PCR were as follows: denaturation at 94°C for 2 min, followed by 25 cycles of 94°C, 30 sec; 57.2°C, 30 sec; and 68°C, 3 min, followed by a final extension at 68°C for 10 min. Thermocycling conditions for the *Opener Primers* (Table 1) in the second PCR were as follows: denaturation at 94°C for 2min followed by 25 cycles of 94°C, 30 sec; 53.1°C, 30 sec; and 68°C, 2 min, followed by a final extension at 68°C for 10 min. PCR products (*Starter and Intermediate DNA*) were re-circularized by ligation with T4 DNA ligase for 15 min at room temperature followed by an optional enzyme inactivation at 65°C for 10 min.

The final product (*Linear Modified DNA*) and the parent plasmid were digested with *NsiI* and *SphI* (New England Biolabs) and then ligated overnight at 14°C.

The final ligation reaction was diluted 1:5 in distilled water and 5 μl was used to transform 50 μl DB3.1 *E*. *coli* chemically competent cells (Invitrogen). Ampicillin resistant colonies were selected for plasmid isolation using the Qiagen MiniPrep Kit (Qiagen, Hilden, Germany). Forward and reverse primers were designed about 250 bp apart using Primer Select (Lasergene-DNAStar, Madison, WI) to perform a primer walk of the entire original and mutagenized plasmids. Plates were sequenced at Boston Children’s Hospital, Boston, MA. Sequencing the entire cDNA of muscular dystrophy was performed the DNA sequencing facility at Nationwide Children’s Hospital, Columbus, OH.

### Deletion of two open reading frames from the RSV plasmid

*Starter Primers* (Table 1), the RSV replicon plasmid MP312, and VENT polymerase (New England Biolabs, Ipswich, MA) were used to generate the *Starter DNA*, under the following thermocycling conditions: denaturation at 94°C for 2 min, followed by 25 cycles of 94°C, 20 sec; 53°C 30 sec; and 72°C, 3 min, followed by 72°C for 5.5 min. Following ligation with T4 DNA ligase, *Opener Primer* pairs (Table 1) and *Pfu Turbo* polymerase (Stratagene) were used to generate the three *Intermediate DNAs* under these conditions: denaturation at 95° for 2 min followed by 25 cycles of 95°C, 20 sec; 51°C, 30 sec; and 72°C, 3 min, followed by 72°C, 10 min, and finally 4°C. The third PCR also used the *Starter Primers* and *Pfu Turbo* polymerase under the thermocycling conditions of the first PCR. Restriction digestion of the parent plasmid and the *Linear Modified DNAs* were done using enzymes *XhoI* (New England Biolabs) and *AarI* (Fermentas, Glen Burnie, MD).

Ligations were performed with T4 DNA Ligase (Invitrogen, Carlsbad, CA) overnight at 14°C. The final ligation reaction was diluted 1:5 in distilled water and 5 μl was used to transform 20 μl of ElectroMax DH10B *E*. *coli* cells (Invitrogen). Electroporation was carried out using BioRad Gene Pulser II (BioRad, Hercules, CA) under the following conditions: 1.8 kV, 25 Faradays and 100 Ω in 0.1-cm cuvettes cooled on ice then 1 ml SOC media was added and cells were incubated 1 hour at 37°C. 200 μl of recovered cells were spread on LB agar plates supplemented with 10 μg/ml tetracycline and incubated for 48 h at 33°C. LB agar plates were prepared by mixing 10g Bacto-tryptone, 5g yeast extract, 10g NaCl and 15g Bacto agar in 1 liter of Nanopure water and autoclaved, then cooled to 50°C before pouring into perti dishes.

Individual colonies were screened for inclusion of the proper ΔM2 fragment by PCR with the *Starter Primers* followed by DNA sequencing to confirm insertion of the correct mutation.
